# Proteomic analysis of the medicinal plant *Artemisia annua*: Data from leaf and trichome extracts

**DOI:** 10.1016/j.dib.2016.02.038

**Published:** 2016-02-23

**Authors:** Laura Bryant, Chhaya Patole, Rainer Cramer

**Affiliations:** Department of Chemistry, University of Reading, Whiteknights, Reading RG6 6AD, UK

**Keywords:** Proteomics, Mass spectrometry, LC–MS/MS, Artemisia annua, Artemisinin, Sequence databases, Proteogenomics, Biosynthesis

## Abstract

This article contains raw and processed data related to research published by Bryant *et al.*[1]. Data was obtained by MS-based proteomics, analysing trichome-enriched, trichome-depleted and whole leaf samples taken from the medicinal plant *Artemisia annua* and searching the acquired MS/MS data against a recently published contig database [2] and other genomic and proteomic sequence databases for comparison. The processed data shows that an order-of-magnitude more proteins have been identified from trichome-enriched *Artemisia annua* samples in comparison to previously published data. Proteins known to have a role in the biosynthesis of artemisinin and other highly abundant proteins were found which imply additional enzymatically driven processes occurring within the trichomes that are significant for the biosynthesis of artemisinin.

**Specifications table**

**Table:** 

Subject area	Proteomics
More specific subject area	Plant proteomics
Type of data	LC-ESI MS/MS data tables and electron microscopy images
How data was acquired	Raw data by nanoUHLPC-MS/MS and ESEM.
Processed MS/MS data by sequence database searching.
Data format	Mascot.mgf files, also deposited as MS/MS raw files
Experimental factors	Trichomes were isolated from frozen leaves of *Artemisia annua* cultivar Artemis. Proteins were extracted and digested from trichome-enriched, trichome-depleted and whole leaf samples.
Experimental features	NanoUHPLC-MS/MS-based proteomics was applied to the analysis of trichome-enriched, trichome-depleted and whole leaf samples of *Artemisia annua*. Protein identification searches were performed against genomic and proteomic sequence databases for comparison of results using Mascot Daemon software. Obtained emPAI values were used for quantitative protein abundance comparisons.
Data source location	EMBL-EBI, Wellcome Trust Genome Campus, Hinxton, Cambridgeshire, CB10 1 SD, UK and Department of Chemistry, University of Reading, Reading, Berkshire, RG6 6AD, UK
Data accessibility	Via the PRIDE (Proteomics Identifications Database) repository at the European Bioinformatics Institute (http://www.ebi.ac.uk/pride/), PXD000703.

**Value of the data**

• The data provides the so far largest set of proteomic data for *A. annua* and thus additional information for studies in need of *A. annua* proteomic data.
• In particular, the data provides a more substantial collection of trichome-specific proteome data for *A. annua*.
• It also provides the basis for a clear demonstration that the number of true protein identifications can be increased alongside a reduction in the number of false positives by utilising an organism-specific database that has undergone comprehensive curation and therefore contains longer contig sequences.
• A large number of proteins that are known to be involved in the biosynthetic pathway to the anti-malarial pro-drug artemisinin were found, such as amorpha-4,11-diene synthase (ADS), cytochrome P450 (CYP71AV1), artemisinic aldehyde *Δ*^11,13^ reductase (DBR2) and aldehyde dehydrogenase 1 (ALDH1).

## 1 Data, experimental design, materials and methods

### 1.1 Solvents and solutions

Solvents were of HPLC-grade and bought from Sigma-Aldrich, Poole, UK, except for water, which was purchased from Fisher Scientific, Loughborough, UK. The isolation buffer contained 200 mM sorbitol (Fluka Biochemika, Buchs, Switzerland), 2 mM sucrose (Sigma-Aldrich), 5 mM succinic acid (Sigma-Aldrich), 5 mM dithiothreitol (Sigma-Aldrich), 1 mM ethylene glycol bis(2-aminoethyl ether)-N,N,N׳,N׳-tetraacetic acid (Sigma-Aldrich), 0.5 mM Na_2_HPO_4_ (Sigma-Aldrich), 0.1 mM Na_4_P_2_O_7_ (Sigma-Aldrich), 25 mM HEPES (Sigma-Aldrich) and 5 mM MgCl_2_ (Sigma-Aldrich) in water. The precipitation solution consisted of 10% (w/v) trichloroacetic acid (Fiedel-de Haen, Buchs, Switzerland) and 0.07% (w/v) 2-mercaptoethanol (Sigma-Aldrich) in cold acetone (Sigma-Aldrich) while the rinsing solution contained 0.07% (w/v) 2-mercaptoethanol in cold acetone. The solubilisation solution was made up of 7 M urea (Sigma-Aldrich) and 2 M thiourea (Sigma-Aldrich) in water.

### 1.2 Plant material

Leaves of *Artemisia annua* field cultivar Artemis (seed source: Mediplant, Switzerland) which had no prior treatment were harvested and frozen at −80 °C.

### 1.3 Isolation of glandular trichomes

**Fig. 1:**
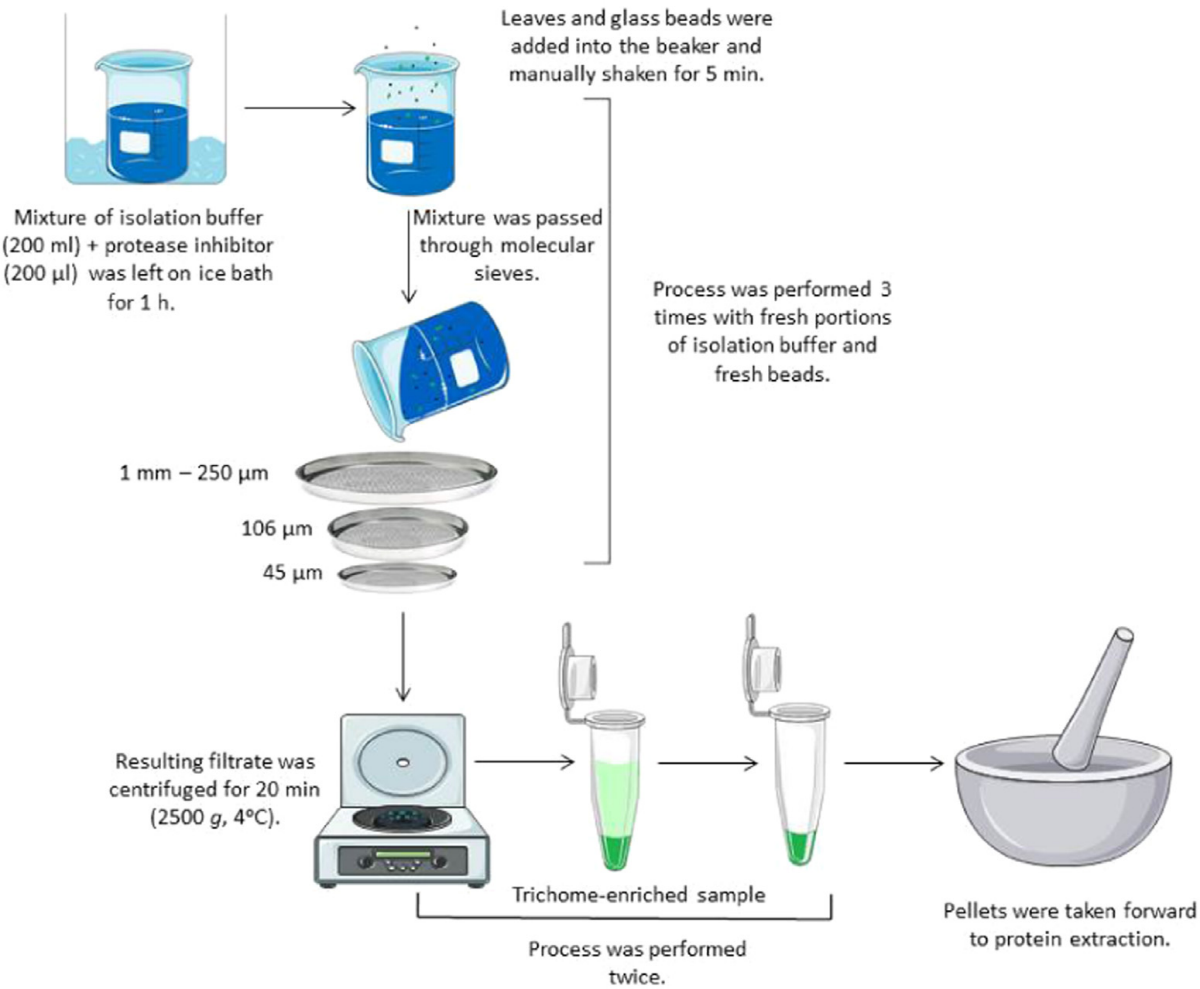
Schematic representation of the isolation of trichomes from frozen *Artemisia annua* leaves using a manual abrasion technique.

A solution containing 200 ml of isolation buffer and 200 μl of protease inhibitor (Calbiochem, Nottingham, UK) was left to stand on ice in a 500-ml bottle for 1 h. Then, 20 g of frozen *Artemisia annua* leaves and 20 g of glass beads (0.5 mm diameter) (Thistle Scientific, Glasgow, Scotland) were added and the bottle was shaken for 5 min. The mixture was passed consecutively through 1-mm, 250-μm, 106-μm and 45-μm molecular sieves (Endecotts, London, UK). Nitrogen gas was used to force the liquid through the 106-μm and 45-μm sieves. The plant material was returned to the 500-ml bottle and the above process was repeated a further two times with each repeat using fresh 200-ml portions of isolation buffer and fresh beads. The combined filtrates were divided into 50-ml tubes and centrifuged for 20 min at 2500 g and 4 °C. The supernatant was disposed of and the pellet divided into four 1.5-ml microcentrifuge tubes and centrifuged for a further 20 min at 2500 g and 4 °C. After discarding the supernatant, the resulting pellets (each ~0.2 g) were used as the enriched glandular trichome sample. Leaf material retained on the 1-mm sieve formed the glandular trichome-depleted sample. See Fig. 1 for a schematic representation of the isolation of the trichomes.

### 1.4 Environmental scanning electron microscopy

**Fig. 2:**
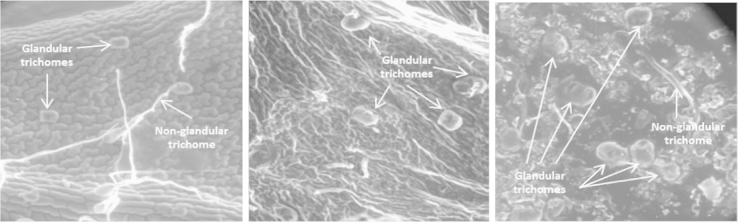
ESEM images of a fresh *Artemisia annua* leaf before abrasion (left),׳trichome-depleted׳ material from the 1-mm sieve (middle) and׳trichome-enriched׳ material (right) post abrasion (adapted from [1]).

ESEM images were taken on a Quanta 600F instrument (FEI, Hillsboro, OR, USA) for plant material which had been caught on the 1-mm sieve and dried. The results were compared against ESEM images of dried enriched glandular trichome isolate (Fig. 2).

### 1.5 Protein extraction

Glandular trichome-enriched pellets were separately crushed using a micro-pestle. Portions of 2 g of the frozen whole leaf material (*i.e.* material from leaves which did not undergo the trichome isolation procedure) and 2 g of glandular trichome–depleted leaf material were separately flash frozen using liquid nitrogen, and ground to a fine powder using a pestle and mortar. Precipitation solution was then added to the samples (1.5 ml to the trichome-enriched samples and 18 ml to the whole leaf and the trichome-depleted samples). All samples were then vortexed and kept at −20 °C for 1 h. Subsequently, the samples were centrifuged for 10 min (4 °C) at 10,000 *g* (trichome-enriched samples) and 4000 *g* (whole leaf and trichome-depleted samples), respectively. The resulting pellets were dissolved in rinsing solution (1.5 ml for trichome-enriched samples and 18 ml for whole leaf and trichome-depleted samples) and kept at −20 °C for 1 h. The samples were then centrifuged for 10 min (4 °C) at 10,000 *g* (trichome-enriched samples) and 4000 *g* (whole leaf and trichome-depleted samples), respectively, and the supernatants were discarded. The steps of adding rinsing solution, centrifugation and discarding of the supernatant were repeated twice, keeping the resulting pellets from each procedure. The trichome-enriched pellets were placed under vacuum for 30 min to dry, and each pellet was dissolved in 200 μl of solubilisation solution, vortexed and centrifuged for 10 min (25 °C) at 10,000 *g*. The resulting supernatants were kept and the pellets were discarded. Similarly, the trichome-depleted and whole leaf pellets were left for 1 h to dry and each pellet was dissolved in 3 ml of solubilisation solution. The sample solutions were then thoroughly vortexed and centrifuged for 10 min (25 °C) at 4000 *g* and the supernatants were retained. Bradford assays were performed to determine the amount of protein in each sample.

### 1.6 Protein digestion

Approximately 150 µg of protein from each (trichome-enriched, trichome-depleted and whole leaf) material were separately digested. A 100-mM dithiothreitol solution (Sigma-Aldrich) was added to the three samples in order to obtain a final concentration of 10 mM dithiothreitol. Each extract was then thoroughly vortexed and kept at 45 °C for 45 min. After being left to cool at room temperature for 5 min, a solution of 90 mM iodoacetamide (Sigma-Aldrich) was added in order to achieve a final concentration of 30 mM of iodoacetamide. A final approximate concentration of 2 M of urea was obtained by diluting each extract with a 50-mM solution of ammonium bicarbonate (Sigma-Aldrich). All extracts were thoroughly vortexed and kept at room temperature in the dark (~45 min). Next, pH strips confirmed that the pH of each extract was between 7.5 and 8 and sequence-grade trypsin (Promega, Southampton, UK) was added using a stock solution of 200 ng/ml. At a protein-to-trypsin ratio of 100:1 all extracts were thoroughly mixed and kept at 37 °C overnight. The next day 0.1% trifluoroacetic acid (TFA; Sigma-Aldrich) was used to stop the digestions.

### 1.7 LC–MS/MS analysis

All digested samples were diluted to a third of their original concentration with a solution of 0.1% TFA. Each sample was then analysed in triplicate on a nanoUHPLC-MS/MS system, similar to previously published protocols [3], [4]. For nanoUHPLC, an UltiMate 3000RSLCnano (Dionex/Thermo Scientific, Hemel Hempstead, UK) was employed with an Acclaim PepMap100 C18 100 μm ×2 cm column as trap column (Dionex/ Thermo Scientific) and an Acclaim PepMap C18 75 μm ×25 cm column as the analytical column (Dionex/ Thermo Scientific), which was maintained at 40 °C. A flow rate of 0.3 µl/min was used to separate the samples using a linear gradient elution with 0.1% formic acid as solution A and 80% acetonitrile/0.1% formic acid as solution B for the mobile phases. The gradient was as follows: 4% B at 0–4 min, 7% B at 5 min, 50% B at 120 min, 90% B at 150–160 min, 4% B at 160–200 min.

MS/MS data was acquired using an LTQ Orbitrap XL mass spectrometer (Thermo Scientific) with the automatic gain control target value set to 500,000 over 500 ms for the orbitrap and 10,000 over 200 ms for the ion trap. MS spectra were acquired at a resolution of 60,000 using the orbitrap mass analyser for m/z 400–2000 scans. For each MS scan, the 5 most intense multiply charged ions were sequentially isolated based on their signal intensity (highest signal intensity first) with a signal threshold set to 5000 and an isolation window of m/z 3. The isolated ions were fragmented in the linear ion trap using collision-induced dissociation (CID). The CID settings included: normalised collision energy: 35%, activation *q* value: 0.25, activation time: 30 ms. Fragment ions were detected over the m/z range of 100–2000. Redundant sequencing was minimised by enabling dynamic exclusion. MS peaks which occurred twice or more times in 30 s were omitted from selection for fragmentation for 60 s. The exclusion list had a restriction of 500 entries.

### 1.8 Data processing, mining and analysis

Raw LC–MS/MS data of each technical replicate for each sample type (trichome-enriched, trichome-depleted and whole leaf sample) were converted into Mascot Generic Files (.mgf files) using Mascot Distiller software (Version 2.3.2; Matrix Science, London, UK). The latter files are available in their compressed form in the supplementary data. Mascot Daemon (Matrix Science) was used to perform searches against sequence databases, combining the results of all three technical replicates of each sample type. Databases included in the Mascot (server version 4.2) searches were: the UniprotKB database (downloaded on 24. April 2012), the NCBInr database (downloaded on 07. June 2012), an in-house contaminants database, the York *A. annua* (Artemis) contig and the recently published trichome Trinity contig database. Searches were performed against the contaminants database in order to evaluate the sample contamination level and screen for contaminants such as keratins and common protein standards that are used in the lab. The York *A. annua* (Artemis) contig database was downloaded from the NCBI website http://www.ncbi.nlm.nih.gov/bioproject/39657 in January 2012 and consists of 116,303 RNA sequences from the cultivar Artemis. It was created at the University of York as part of a transcriptome shotgun assembly project [2]. The trichome Trinity contig database was available through Soertaert *et al.* [5]. The following parameters were applied to each search: peptide mass tolerance: 10 ppm; MS/MS tolerance: 0.8 Da; peptide charge: +2, +3, +4; missed cleavages: 2; fixed modification: carbamidomethyl (C); variable modification: Oxidation (M); and enzyme: trypsin. Searches were performed against the *A. annua* contig and UniprotKB databases using *viridplantae* as the taxonomy.

The databases ׳York Artemis contigs׳, ׳Artemisia trichome Trinity contigs׳, ׳NCBInr *viridiplantae*׳, ׳Uniprot *A. Annua*׳ and ׳Uniprot *viridiplantae*׳ were compared against each other based on protein identification search results from the MS/MS data of the trichome-enriched *Artemisia Annua* sample material (Table in supplementary data, adapted from [1]).

Mascot-derived emPAI values were applied as a way of comparing the merged database results from the trichome-enriched sample to the merged database results from the trichome-depleted sample and whole leaf sample, using Mascot searches against the ׳York Artemis contigs׳ database. The proportional fold difference for each contig/protein was calculated by dividing the emPAI value of the trichome-enriched sample with the emPAI value of the trichome-depleted and whole leaf sample, respectively. To gain the emPAI values the Mascot search results were first submitted to the built-in Percolator software. An ׳expect cut-off׳ of 0.05 was applied and a filter of at least 2 ׳significant sequences׳ was applied to the Report Builder within Mascot when exporting the Mascot search results as .csv files. For each comparison two lists were created: one for proteins/contigs that were present in both sample types in all triplicates and one for proteins/contigs that were only present in all triplicates of one sample type but not in the other. The proportional difference in emPAI value was used to rank the proteins/contigs in the first list, while the absolute emPAI value was used to rank the proteins/contigs in the second list (see results published in [1]).

**Fig. 3:**
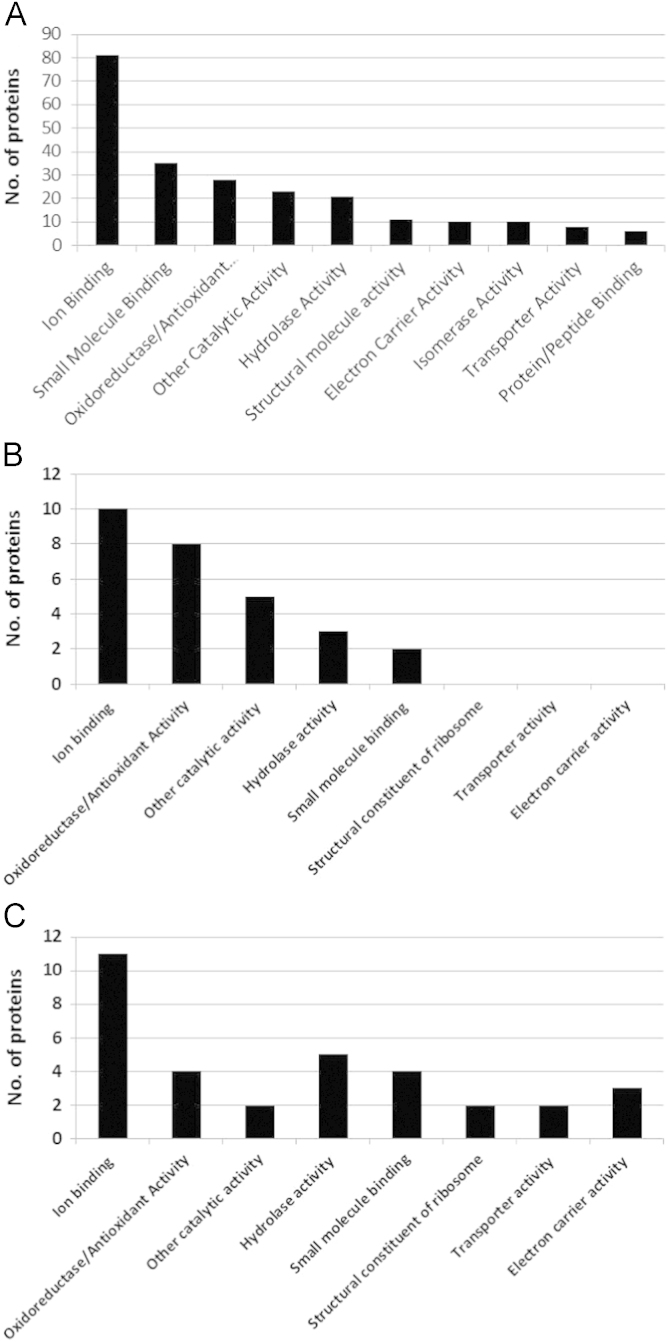
Functional classifications (according to GO terms) of the trichome-enriched sample proteins identified from Mascot searches against the (A) Uniprot *viridiplantae*׳ database, (B) ׳York Artemis contigs׳ database for protein identifications with higher abundance in trichome-enriched sample material, and (C) ׳York Artemis contigs׳ database for protein identifications with lower abundance in trichome-enriched sample material. Mascot search results were submitted to Percolator with an ׳expect cut-off׳ threshold of 0.05 and filtered using a minimum number of significant sequences of 2. Adapted from [1].

Due to the lack of annotation in the ׳York Artemis contigs׳ database, contigs were translated into known protein homologues using BLASTx searches with an E-value cut-off of 0.01, non-redundant protein sequences specified as the database and Artemisia as the organism. Functions of the resultant proteins were confirmed using Uniprot Protein KnowledgeBase. BLASTx searches were performed against contigs with the highest ranking proportional emPAI values or absolute emPAI values from the comparisons outlined above. Proteins identified from the BLASTx searches with associated gene ontology (GO) molecular function terms were divided into different categories based on the UniProt database terminology (http://www.uniprot.org). Similarly, proteins identified from Mascot searches against the ׳Uniprot *viridiplantae*׳ database were divided into different categories according to their UniProt GO molecular function terms. Fig. 3 displays the classifications into these categories. A number of proteins fell into more than one GO category, depending on their (multiple) GO annotation.

MS proteomics data have also been deposited as Mascot.dat files to the ProteomeXchange Consortium (http://proteomecentral.proteomexchange.org) via the Proteomics Identifications Database (PRIDE) partner repository at the European Bioinformatics Institute (http://www.ebi.ac.uk/pride/), PXD000703.

## Conflict of interest

None.

## Appendix A Supplementary material

Supplementary material

Supplementary material **Data files** Compressed data files of the three technical nanoHPLC-ESI MS/MS analysis replicates for each sample type (trichome-enriched, trichome-depleted and whole leaf sample). The data files are Mascot Generic Files (.mgf files) which were obtained by converting the raw MS/MS files using Mascot Distiller software (Version 2.3.2; Matrix Science, London, UK).
