# Supplementary material for: Proteomic analysis of the medicinal plant Artemisia annua: Data from leaf and trichome extracts
Source: Data Brief. 2016 Feb 23;7:325–31. doi: 10.1016/j.dib.2016.02.038 (PMC4781977; doi:10.1016/j.dib.2016.02.038)
Supplement: Supplementary file 1 — Supplementary material [file mmc1.docx]

Conflicts of interest: none.
