# Supplementary material for: Proteomic analysis of the medicinal plant Artemisia annua: Data from leaf and trichome extracts
Source: Data Brief. 2016 Feb 23;7:325–31. doi: 10.1016/j.dib.2016.02.038 (PMC4781977; doi:10.1016/j.dib.2016.02.038)
Supplement: Supplementary file 2 — Supplementary material Data files Compressed data files of the three technical nanoHPLC-ESI MS/MS analysis replicates for each sample type (trichome-enriched, trichome-depleted and whole leaf sample). The data files are Mascot Generic Files (.mgf files) which were obtained by converting the raw MS/MS files using Mascot Distiller software (Version 2.3.2; Matrix Science, London, UK). [file mmc2.zip › MGF files + supplementary table/Supplementary Table.docx]

**Table - Protein identification search results for the MS/MS data of the trichome-enriched *A. annua* sample material (adapted from [1])**

| Target database | # of protein family hits | # of peptides matched above identity threshold in target database | # of peptides matched above identity threshold in decoy database | FDR |
| --- | --- | --- | --- | --- |
| York Artemis contigs  (116,303 sequences) | 671 | 3072 | 58 | 1.9% |
| Artemisia trichome Trinity contigs (150,282 sequences) | 684 | 2636 | 85 | 3.2% |
| NCBInr *viridiplantae*  (1,079,491 sequences) | 419 | 1286 | 60 | 4.7% |
| UniProt *A. annua*  (118 sequences) | 17 | 179 | 11 | 6.2% |
| UniProt *viridiplantae*  (32,329 sequences) | 319 | 1271 | 112 | 8.8% |
